# SLC30A3 Responds to Glucose- and Zinc Variations in ß-Cells and Is Critical for Insulin Production and *In Vivo* Glucose-Metabolism During ß-Cell Stress

**DOI:** 10.1371/journal.pone.0005684

**Published:** 2009-05-25

**Authors:** Kamille Smidt, Niels Jessen, Andreas Brønden Petersen, Agnete Larsen, Nils Magnusson, Johanne Bruun Jeppesen, Meredin Stoltenberg, Janetta G. Culvenor, Andrew Tsatsanis, Birgitte Brock, Ole Schmitz, Lise Wogensen, Ashley I. Bush, Jørgen Rungby

**Affiliations:** 1 Department of Pharmacology, University of Aarhus, Århus, Denmark; 2 Department of Neurobiology, Institute of Anatomy, University of Aarhus, Århus, Denmark; 3 Department of Endocrinology and Metabolism C, Aarhus University Hospital, Århus, Denmark; 4 Research Laboratory for Biochemical Pathology, Aarhus University Hospital, Århus, Denmark; 5 Department of Endocrinology M, Aarhus University Hospital, Århus, Denmark; 6 Department of Pathology, University of Melbourne, Melbourne, Victoria, Australia; 7 Oxidation Biology Laboratory, Mental Health Research Institute, Parkville, Victoria, Australia; University of Bremen, Germany

## Abstract

**Background:**

Ion transporters of the Slc30A- (ZnT-) family regulate zinc fluxes into sub-cellular compartments. β-cells depend on zinc for both insulin crystallization and regulation of cell mass.

**Methodology/Principal Findings:**

This study examined: the effect of glucose and zinc chelation on ZnT gene and protein levels and apoptosis in β-cells and pancreatic islets, the effects of ZnT-3 knock-down on insulin secretion in a β-cell line and ZnT-3 knock-out on glucose metabolism in mice during streptozotocin-induced β-cell stress. In INS-1E cells 2 mM glucose down-regulated ZnT-3 and up-regulated ZnT-5 expression relative to 5 mM. 16 mM glucose increased ZnT-3 and decreased ZnT-8 expression. Zinc chelation by DEDTC lowered INS-1E insulin content and insulin expression. Furthermore, zinc depletion increased ZnT-3- and decreased ZnT-8 gene expression whereas the amount of ZnT-3 protein in the cells was decreased. Zinc depletion and high glucose induced apoptosis and necrosis in INS-1E cells. The most responsive zinc transporter, ZnT-3, was investigated further; by immunohistochemistry and western blotting ZnT-3 was demonstrated in INS-1E cells. 44% knock-down of ZnT-3 by siRNA transfection in INS-1E cells decreased insulin expression and secretion. Streptozotocin-treated mice had higher glucose levels after ZnT-3 knock-out, particularly in overt diabetic animals.

**Conclusion/Significance:**

Zinc transporting proteins in β-cells respond to variations in glucose and zinc levels. ZnT-3, which is pivotal in the development of cellular changes as also seen in type 2 diabetes (e.g. amyloidosis in Alzheimer's disease) but not previously described in β-cells, is present in this cell type, up-regulated by glucose in a concentration dependent manner and up-regulated by zinc depletion which by contrast decreased ZnT-3 protein levels. Knock-down of the ZnT-3 gene lowers insulin secretion *in vitro* and affects *in vivo* glucose metabolism after streptozotocin treatment.

## Introduction

The in- and efflux of zinc ions in cells has a number of physiological roles. Apart from being an essential metal for many enzymatic reactions [1], zinc is necessary in β-cells for proper storage of insulin by crystallization [2]. Furthermore, zinc, which is co-secreted with insulin, has paracrine effects on glucagon secretion from neighbouring α-cells [3], [4] and participates in the regulation of β-cell mass by inducing cell death in a dose-dependent manner by largely unknown mechanisms [5]–[7]. Recently, mutations in the gene for a β-cell zinc-transporter have been identified as major risk mediators for type 2 diabetes and a zinc transporter appears to be responsible for a substantial part of the β-cell autoimmunity in type 1 diabetes suggesting a key role of β-cell zinc metabolism in these conditions [8]–[12].

Zinc and zinc transporters are important in other cell types and diseases, particularly in degenerative amyloidosis caused by the formation of β-amyloid plaques in the central nervous system. Such changes cause neuronal degeneration, resulting in Alzheimers disease (AD). Endogenous metals like zinc contribute to the aggregation of β-amyloid plaques and knocking out zinc transporters (ZnT-3) in mice leads to significantly fewer and smaller plaques and lower zinc concentrations in the hippocampus [13], [14]. It has been hypothesised that intracellular chelatable zinc as well as extracellular zinc contribute to apoptosis [15].

Two different zinc-transporter families regulate zinc trafficking in cells. Efflux from the cytoplasm to extracellular spaces and intracellular membrane-limited compartments is mediated by ZnT proteins (SLC30A-family). Conversely, influx is regulated by ZIP proteins (SLC39A-family). So far, at least 10 ZnTs and 14 ZIPs have been identified in mammals and zinc transport activity has been confirmed with 7 ZnTs and 9 ZIPs [16].

The distribution of individual zinc transporters in the pancreas appears to vary. In mouse pancreas the acinar cells express high levels of ZnT-1 and ZnT-2, this level is reduced in zinc-depletion states [17]. ZnT-3 is uninvestigated in pancreas, but ZnT-3^−/−^ mice have histochemically reactive zinc in pancreatic islet cells [18] which indicates that other members of the ZnT family contribute to the compartmentalization of zinc into secretory granules. ZnT-4 expression is present in pancreatic islets but the distribution of the ZnT-4 protein is unknown [19]. ZnT-5 is abundantly expressed in pancreatic β-cells and the localisation of ZnT-5 in secretory granules suggests that ZnT-5 is involved in supplementing zinc for the formation and stabilization of insulin-zinc complexes [20]. No functional studies, however, have confirmed the role of ZnT-5. ZnT-6 and ZnT-7 are expressed in acinar cells from mouse pancreata [17]. The ZnT-8 gene is highly expressed in β-cells. ZnT-8 is localised in secretory granules which indicates a role for ZnT-8 similar to ZnT-5 in providing zinc for insulin storage [21], [22]. Recently it was found that ZnT-8 auto-antibodies are present in 60–80% of new-onset Type 1 diabetes patients [9], and that a polymorphism in the ZnT-8 gene leads to impaired pro-insulin conversion and is associated with Type 2 diabetes [8], [23]. We recently demonstrated that ZnT-8 is expressed at high levels also in human adipose tissue (high levels in subcutaneous fat from lean, low levels in intra-abdominal fat from obese) [24]. ZnT-9 and ZnT-10 have not yet been described in pancreatic cells.

The molecular events responsible for insulin processing in β-cells thus depend on the regulatory processes for zinc metabolism. Zinc has a number of important paracrine effects in islets and a zinc transporter (ZnT-8) seems to be critical for the genetic risk of diabetes. Therefore, we hypothesized that the regulation of ZnT gene expression is actively controlled by changes in glucose and zinc concentration, and that genetic manipulation of ZnTs may influence *in vivo* glucose homeostasis.

In order to determine whether this system is influenced by glucose- and/or zinc levels in β-cells we examined the expression patterns of ZnT-1 and ZnT-3-8 in glucose sensitive INS-1E cells at different glucose concentrations and the expression patterns of ZnT-3 and ZnT-8 during zinc-depletion. In mouse islets ZnT-3 and ZnT-8 expression was examined at different glucose concentrations. The apoptotic and necrotic status of the INS-1E cells at different glucose levels and after zinc chelation was tested by measuring the expression patterns of the anti-apoptotic gene, B-cell leukemia/lymphoma 2 (Bcl-2), and the pro-apoptotic gene, Bcl-2-associated X protein (Bax) and by measuring DNA fragmentation. In mouse islets the effect of different glucose concentrations on the Bax/Bcl-2 ratio was analysed. In addition, we developed a specific anti-ZnT3 antibody and described the presence of ZnT-3 protein in INS-1E cells and mouse islets. Further, since ZnT-3 appears to be particularly responsive to variations in glucose, we investigated the effects of ZnT-3 knock-down in INS-1E on *in vitro* insulin secretion and ZnT-3 knock-out in mice on *in vivo* glucose metabolism during streptozotocin-induced β-cell stress.

## Materials and Methods

### Cell cultures

INS-1E cells were cultured in a 5% CO_2_ atmosphere in complete RPMI 1640 supplemented with 11 mM glucose, 10% (v/v) heat-inactivated fetal bovine serum, 50 µM β-mercaptoethanol, 2 mM L-Glutamine, 100 U/ml penicillin, and 100 µg/ml streptomycin. For stimulation assays INS-1E cells were plated (1,400,000 cells/well) into 6-well plates (NUNC) at 11 mM glucose and cultured for 24 hours. Then cells were stimulated for 24 hours with 2, 5 or 16 mM glucose in replicas of 9 followed by RNA extraction. DiEthylDiThioCarbamate (DEDTC) treatments were performed with +/−100 µM DEDTC (Merck, Darmstadt, Germany) dissolved in complete RPMI 1640 medium with 5 mM glucose in replicas of 6 for RNA extraction and replicas of 3 for insulin measurements. Controls were stimulated for 24 hours with RPMI 1640 medium and 5 mM glucose in replicas of 6 and 3 for RNA extraction and insulin measurements, respectively.

### Harvest and stimulation of islets

4 week old male BALB/CA mice were killed by cervical dislocation. For gene expression analysis islets were split into groups of 60, cultured for 24 hours in a 5% CO_2_ atmosphere in RPMI 1640 supplemented with 100 U/ml penicillin, and 100 µg/ml streptomycin. Then cells were washed in PBS and stimulated with RPMI 1640 supplemented with 100 U/ml penicillin, and 100 µg/ml streptomycin, 10% (v/v) heat-inactivated fetal bovine serum, 2 mM L-Glutamine, 2, 5 or 16 mM glucose for 24 hours in replicas of 6 followed by RNA extraction. For western blotting analysis 200 islets were stimulated with 100 µM DEDTC dissolved in RPMI 1640 medium supplemented with 100 U/ml penicillin, and 100 µg/ml streptomycin, 10% (v/v) heat-inactivated fetal bovine serum, 2 mM L-Glutamine with 10% FBS, 2 mM L-Glutamine, and 5 mM glucose for 24 hours followed by cell lysis.

### RNA extraction and cDNA synthesis

INS-1E RNA was extracted using Qiagen RNeasy Mini Kit (VWR, Denmark) and treated with DNase (VWR, Denmark). The RNA quality was controlled on a 1% (v/v) agarose gel stained with ethidium-bromide. 500 ng total RNA was reversely transcribed using ImProm-II™ Reverse Transcription System (Promega, Denmark) and oligo dT_18_ primers (TAC, Copenhagen). The cDNA was checked for genomic DNA contamination by PCR analysis using Qiagen HotStarTaq Master Mix Kit (VWR, Denmark) with an intron-spanning primer-set of β-actin (TAC, Copenhagen). The PCR product was analysed by ethidium bromide staining after electrophoresis in a 1% agarose gel.

### RNA amplification

RNA from islet was extracted followed by RNA amplification using RiboAmp® HS^PLUS^ Kit (MDS Analytical Technologies) which, in two rounds, leads to a linear amplification of mRNA from total RNA. 500 ng RNA was reversely transcribed using ImProm-II™ Reverse Transcription System (Promega, Denmark) and used for analysis of ZnT-8, insulin, Bax and Bcl-2 expression whereas 2 mg RNA was used for analysis of ZnT-3.

### Real-time PCR

Quantitative real-time PCR was performed in duplicate with IQ Sybr Green supermix (Bio-Rad, Denmark) in a MyiQ Single-Color Real-Time PCR Detection System (Bio-Rad, Denmark). For all reactions a melting curve was included. The results were analysed with iQ™5 Optical System Software, Version 2.0. Starting quantities were calculated from a standard curve. Values were normalised to the geomean of three housekeeping genes (HKGs) [25]. The expression of the HKGs were analysed to assure stable expression, and neither glucose nor DEDTC treatment seemed to change the gene expression of the selected HKGs (data not shown).

### Primers used for RealTime PCR

#### Rat

Ubiquitin Conjugase-7 (UBC-7): F: 5′CAG CTG GCA GAA CTC AAC AA 3′, R: 5′TTT GGG TGC CAA ATC TCT GT 3′. Annealing temp: 58°C. Hypozanthine-guanine Phosphoribosyltransferase (HPRT): F: 5′GCA GAC TTT GCT TTC CTT GG 3′, R: 5′CCG CTG TCT TTT AGG CTT TG 3′. Annealing temp: 58°C. Cyclophilin A (CycA): F: 5′AGG TCC TGG CAT CTT GTC CA 3′, R: 5′CTT GCT GGT CTT GCC ATT CC 3′. Annealing temp: 58°C. Clathrin (Cltc): F: 5′AAG GAG GCG AAA CTC ACA GA 3′, R: 5′GAG CAG TCA ACA TCC AGC AA 3′. Annealing temp: 59°C. Heat Shock Protein (HSPcb): F: 5′GAT TGA CAT CAT CCC CAA CC 3′, R: 5′CTG CTC ATC ATC GTT GTG CT 3′. Annealing temp: 59°C. ZnT-1: F: 5′CCC AGC TTC ATA CAT GCA GGT G 3′, R: 5′CCT TGC TCT TCT CCC CTA TAT GCT C 3′. Annealing temp: 63°C. ZnT-3: F: 5′TCC TCT TCT CTA TCT GCG CCC 3′, R: 5′TGT GCG GAG GCA ACG TGG TAA 3′. Annealing temp: 59°C. ZnT-4: F: 5′GAT CGG AGA GCT TGT AGG TGG ATA 3′, R: 5′ACA CCA GCA TGA CAC TGA TCA TGG 3′. Annealing temp: 63°C. ZnT-5: F: 5′ATG GCC GAA TAG AAA TTC TC 3′, R: 5′CCT TTC TGT CCT CTT ACC ACT C 3′. Annealing temp: 53°C. ZnT-6: F: 5′GCT GAC CGA AGG TCC TGG AAG A 3′, R: 5′TAG GCA GCG CTA GGT CTC CTC A C 3′. Annealing temp: 65°C. ZnT-7: F: 5′ATG TTG CCC CTG TCC ATC AAG G 3′, R: 5′TCG GAG ATC AAG CCT AGG CAG T 3′. Annealing temp: 60°C. ZnT-8: F. 5′GGT GGA CAT GTT GCT GGG AG 3′, R: 5′CAC CAG TCA CCA CCC AGA TG 3′. Annealing temp: 56°C. Insulin (INS): F: 5′CGC TTC CTG CCC CTG CTG GC 3′, R: 5′CGG GCC TCC ACC CAG CTC CA 3′. Annealing temp: 65°C. Bax: F: 5′GTG AGC GGC TGC TTG TCT 3′, R: 5′GTG GGG GTC CCG AAG TAG 5′. Annealing temp: 60°C. Bcl-2: F: 5′GTA CCT GAA CCG GCA TCT G 5′, R: 5′GGG GCC ATA TAG TTC CAC AA 3′. Annealing temp: 59°C.

#### Mouse

HPRT: F: 5′AAG CTT GCT GGT GAA AAG GA 3′, R: 5′TTG CGC TCA TCT TAG GCT TT 3′. Annealing temp: 57°C. CycA: F: 5′GTG GTC TTT GGG AAG GTG AA 3′, R: 5′TTA CAG GAC ATT GCG AGC AG 3′. Annealing temp: 58°C. UBC-7: F: 5′GGA ACT GGG CTG CAA TAA AA 3′, R: 5′CCG GAT CAT GTT GTG CTA TG 3′. Annealing temp: 58°C.BAX: F: 5′TGC AGA GGA TGA TTG CTG AC 3′, R: 5′GAT CAG CTC GGG CAC TTT AG 5′. Annealing temp: 57°C. BCL-2: F: 5′ AGG AGC AGG TGC CTA CAA GA 3′, R: 5′GCA TTT TCC CAC CAC TGT CT 3′. Annealing temp: 57°C. ZnT-8: F: 5′TTG GTT TTC ATA CGG CTT CC 3′, R: 5′GAT GCA AAG GAC AGA CAG CA 3′. Annealing temp: 57°C. ZnT-3: F: 5′AGC CAT GGA TCT ACA GGT GC 3′, R: 5′CGC AGA TGG AGA AGA GGA AG 3′. Annealing temp: 59°C. Insulin: F: 5′GAC CCT CCA CAC CTA GGA CA 3′, R: 5′AAG CAG CAC CTT TGT GGT TC 3′. Annealing temp: 57°C.

#### Rat and mouse

The intron-spanning primers of β-actin: F: 5′CTA CAA TGA GCT GCG TGT GGC 3′, R: 5′ GTC CAG ACG CAG GAT GGC ATG 3′. Annealing temp: 65°C. cDNA gives a band of 269 basepair, and genomic DNA gives a band of 732 basepair. All PCR sequences were confirmed by sequencing with Thermo Sequenase™ Dye Terminator Cycle Sequencing Premix Kit (Amersham Bioscience, Denmark).

### Insulin measurement

INS-1E cells were treated with 100 µM DEDTC in complete RPMI medium for 24 hours. Hereafter the cells were incubated for 2 hours in a Krebs-Ringer bicarbonate HEPES buffer (KRBH) (at pH 7.4) containing 115 mM NaCl, 4.7 mM KCl, 1.2 mM MgSO_4_, 2.6 mM CaCl_2_, 1.2 mM KH_2_PO_4_, 20 mM HEPES, 5 mM NaHCO_3_, 0.1% (v/v) human serum albumin (HSA) (Sigma, Denmark), +/−100 µM DEDTC and 5 mmol/l glucose for insulin release determination. The incubation medium was collected for insulin analysis and insulin release was measured. Afterwards, the cells were washed in Earle's basal medium (Invitrogen, Denmark) at room temperature before adding 1 ml ice-cold Earle's basal medium in which the cells were scraped off with a rubber policeman. After centrifugation, the medium was discharged and the intact cells re-suspended partly in a buffer containing 0.75% (v/v) glycine and 0.25% (v/v) bovine serum albumin adjusted to pH 8.8 for insulin determination after sonication and centrifugation at 30,000×g for 30 minutes at 4°C, and partly in 0.1 mol/l NaOH for protein determination. Total protein was determined using BCA Protein Assay Reagent Kit from PIERCE, US (Bie & Berntsen A/S, Denmark).

### Insulin assay

Samples of the incubation medium were immediately frozen for insulin analysis. Insulin content was determined using an ultrasentive Rat Insulin Elisa Kit from DRG Diagnostics (VWR. Denmark).

### Cell Death ELISA Assay

Fragmentation of histone-associated-DNA after cell death induced by glucose or DEDTC was determined by photometric enzyme immunoassay (Cell Death Detection ELISA^PLUS^, Roche Applied Science). INS-1E cells were plated on 24-well plate with 200,000 cells per well and stimulated 36 hours later either with 5 mM, 11 mM, 16 mM glucose or 100 µM DEDTC and 5 mM glucose for 24 hours. Experiments were performed in quadruplicates. Cells were scraped of with a rubber policeman and centrifuged at 200×g for 10 minutes. Supernatants containing DNA from necrotic cells were removed and stored at 4°C for further analysis. Cell pellets containing DNA fragments were lysed and centrifuged at 200×g for 10 minutes. The supernatant containing the cytoplasmic fraction and the supernatant containing the DNA from necrotic cells were transferred into streptavidin coated microtiter plate in duplicate and incubated with Anti-histone-biotin. The amount of fragmented DNA bound to Anti-DNA-peroxidase was measured by ABTS (2,2′-azino-bis(3-ethylbenzthiazoline-6-sulphonic acid) at 405 nm and 490 nm as reference wavelength.

### Cell preparation and zinc ion staining

INS-1E cells were grown for 24 hours in staining chambers at a density of 400,000 per chamber. The cells were rinsed in a KRBH buffer supplemented with 0.1% (v/v) HSA and 11 mM glucose as described above, and were then exposed to 0.04% (v/v) sodium sulfide dissolved in KRB+0.1% (v/v) HSA for 60 minutes at room temperature. The cells were rinsed in KRBH and fixed using glutaraldehyde (GA) (3% (v/v), 100 mM phosphate-buffered aqueous solution) at 4°C overnight. The fixed cells were rinsed in distilled water. Slides with cells were placed in jars filled with the AMG developer [19], kept in darkness at 26°C, and developed for 60 minutes. Thereafter slides were placed in 5% (v/v) sodium thiosulfate for 10 minutes in order to stop the AMG development, rinsed in demineralized water for 10 minutes, and air-dried. Finally, the slides were counterstained with 0.1% (v/v) toluidine blue for 90 seconds (for details see e.g. [26]). Control of zinc ion specificity was performed as previously described [26], [26], [27].

### Western blotting

Aliquots of protein from mouse islets, rat brain and INS-1E cells (+/−100 µM DEDTC; mock- or ZnT-3 siRNA transfected) were resolved by sodium dodecyl sulfate-polyacrylamide gel (10% polyacrylamide gels), transferred to nitrocellulose, blocked with 5% non-fat milk in Tris-Buffered Saline Tween-20 (10 mM Tris, 150 mM NaCl, pH 8.0, and 0.1% Tween-20), and incubated with ZnT-3 antibody (1∶2500). β-actin was used as an internal control. The membrane was washed and incubated with horseradish peroxidase-conjugated goat anti-rabbit IgG antibody (1∶4000) (Invitrogen, Denmark) as secondary antibody, and proteins were visualized by ECL Plus Western Blotting Detection System Amersham (GE Healthcare, Denmark).

### ZnT-3 polyclonal antibody

A custom synthesised peptide H-SLRLKSLFTEPSEPLPEEC-OH was used to generate the antibody (Mimotopes). It had an additional cysteine at the C-terminus for coupling to carrier protein. The sequence is to human ZnT-3 in the N-terminal domain where there is strong conservation with mouse ZnT-3. It was coupled at the C-terminus to diphtheria toxoid by Mimotopes for immunization in rabbits with Freund's Adjuvant. Rabbits 04/13 and 04/14 were immunized 9 times at monthly intervals, and bled at 2 weeks after boosts. Serum was incubated at RT for 40 minutes with crushed lyophilised mouse liver to capture any non-specific antibodies. ZnT-3 has a moleculear weight of 44 kDa.

### ZnT-3 immunohistochemistry

The cells (grown in staining chambers) were fixed in 4% (v/v) paraformaldehyde (PFA) for 20 minutes, and subsequently rinsed carefully in TBS for 20 minutes, followed by TBS and triton 1% (v/v) for 10 minutes. The pre-incubation in TBS and 1% (v/v) triton and 0.2% (v/v) milk lasted 30 minutes. The cells were incubated with a ZnT-3 antibody (1∶2000) (kindly provided by Professor R. Palmiter, Seattle, USA) at 4°C overnight, rinsed in TBS and 1% (v/v) triton, and incubated with 10 nm colloidal gold labelled secondary goat anti-rabbit antibody, 1∶50 dilution, (Aurion) for 1 hour at room temperature. After rinsing in TBS followed by water, the cells exposed to silver enhancement for 40 minutes (developer, enhancer, gum arabic; 1∶1∶1) using a commercial kit (Aurion R-GENT SE_LM). Finally, the cells were rinsed in water and counterstained with 0.1% (v/v) toluidine blue. After light microscopy, selected parts were chosen for re-embedding in Epon (see e.g. [28]) and 75 nm sections were cut, placed on grids, and finally counterstained with lead citrate and saturated uranyl acetate. The sections were examined by routine transmission electron microscopy. Cells not incubated with ZnT-3 antibody remained unstained.

### ZnT-3 siRNA transfection in INS-1E cells

For transfection with small interference RNA (siRNA) INS-1E cells were plated in 24-well plates at a density of 100,000 cells per well. 72 hours later the cells were transfected with ZnT-3 or control non-silencing (mock) siRNA in OPTI-MEM with 0.7 µl DharmaFect (Dharmacon) in a total volume of 500 µl. The final siRNA concentration was 100 nM. After 16 hours of transfection the transfection-medium was replaced with complete RPMI medium without antibiotics. Cells were cultured for an additional 48 hours before insulin expression and secretion studies were performed. Target sequence for ZnT-3 siRNA was (J-099172-10), and target sequence for mock was (D-001810-01-20), (Dharmacon). Efficiency of transfection was approximately 40% as assed with RT-PCR. For RNA expression and insulin secretion analysis replicas of 3 and 4 were employed, respectively.

### In vivo glucose metabolism after ZnT-3 knock-out

The study was undertaken in accordance with Danish law and the University of Aarhus guidelines for animal welfare. Every effort was made to minimize the number of animals used and their suffering. To examine whole body glucose metabolism after ZnT-3 knock-out we employed a whole body ZnT-3 knock-out (ZnT-3−/−) mouse model using male animals only [18]. Male, age matched wild-type C57Bl6 (ZnT-3+/+) mice bred from ZnT-3+/− littermates were used as controls. Three months old mice were housed under standard laboratory conditions with free access to food and water. For fasting measurements food was removed for 12 hours maintaining free access to water. For glucose measurements samples were obtained by tail bleeding. For the intraperitoneal glucose tolerance test (IPGTT) 2 g glucose/kg body weight were injected and blood glucose was measured after 15, 30, 60 and 120 minutes. All fasting and non-fasting glucose measurements were performed between 8 AM and 10 AM. For the first experiment freshly dissolved streptozotocin was injected intraperitoneally (50 mg/kg/day) to 15 knock-out and 15 wild-type animals for 5 days and fasting blood glucose levels were measured after a 3 day wash-out. In a smaller group (10 knock-outs and 10 wild-types) this was repeated for 3 weeks with IPGTTs being performed after each series of injections after a 3 day wash-out. Since none of the animals developed diabetes we performed a high-dose streptozotocin experiment (200 mg/kg/day for 3 days) in 10 randomly selected mice (5 knock-outs, 5 wild-types with one accidental death in the WT-group after day 1). On the day after the last injection we measured non-fasting glucose levels and the following day performed an IPGTT. Blood glucose was measured using an OneTouch Ultra meter (Lifescan, Milpitas, USA.).

### Data analysis

Cell cultures: Means are shown ±SEM. Comparisons between groups in RT PCR experiments and analysis of DNA fragmentation were made by Mann-Whitney test. Student unpaired t-test was used to analyse insulin content and secretion data in the DEDTC treatment experiment. ZnT-3 siRNA experiment: AUCs of insulin secretion/insulin content were compared by Mann-Whitney test. IPGTT: AUCs of glucose levels after intraperitaneal exposure were compared by two-way ANOVA.

## Results

### ZnT-3, ZnT-5 and ZnT-8 gene expressions are regulated by glucose

**Figure 1:**
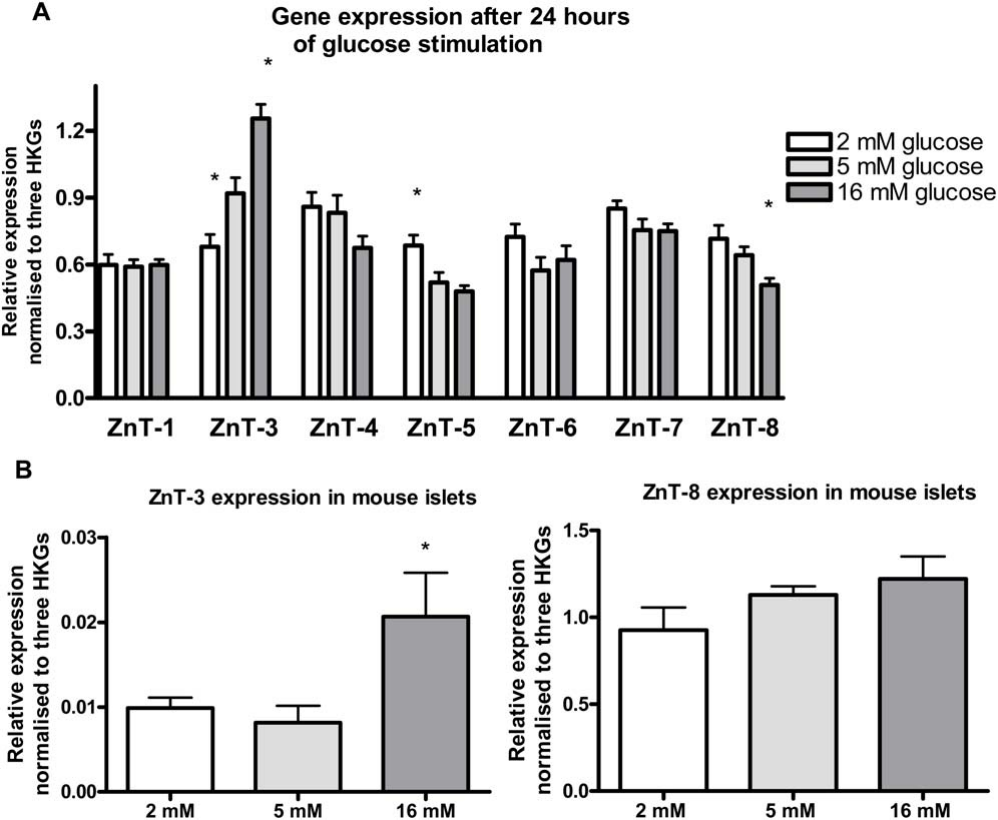
Relative gene expression of ZnTs in INS-1E cells and mouse islets after 24 hours of glucose stimulation. A) INS-1E cells: Stimulations were performed at 2, 5 or 16 mM glucose. Normalised to UBC-7, CycA and β-actin. Data are mean and SEM (*p<0.05). N = 9. B) Mouse islets: Stimulations were performed at 2, 5 or 16 mM glucose. Normalised to UBC-7, CycA and HPRT. Data are mean and SEM (*p<0.05). N = 6.

We cultured INS-1E cells for 24 hours at 2, 5 or 16 mM glucose in order to study the gene expression of selected ZnTs. ZnT-1 and ZnT-3-8 were expressed at all glucose concentrations. ZnT-5 expression was significantly up-regulated at the lowest glucose-concentration (p<0.01) while ZnT-3 was significantly down-regulated (p<0.05). At the highest glucose concentration, ZnT-8 was significantly down-regulated (p<0.05) whereas ZnT-3 was significantly up-regulated (p<0.01) (Fig. 1A). All comparisons are relative to 5 mM glucose. Islets from mice cultured for 24 hours at 2, 5 or 16 mM glucose had significantly higher ZnT-3 expression at 16 mM glucose compared to 2 mM glucose (p<0.05). ZnT-8 expression was not significantly different at the various glucose concentrations (Fig. 1B).

### Zinc chelation stimulates ZnT-3 gene expression but inhibits ZnT-8 and insulin gene expression, and lowers insulin content

**Figure 2:**
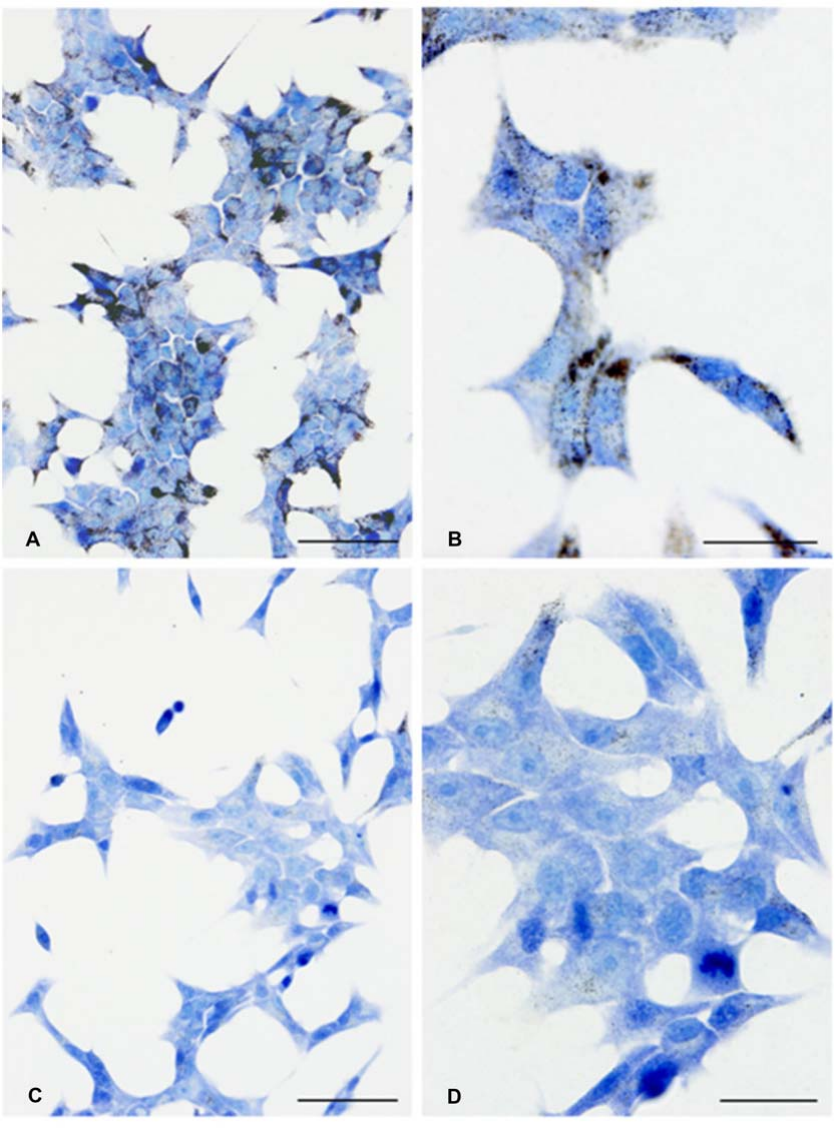
Zinc content of INS-1E cells. (A+B) Zn^2+^ autometallography of untreated INS-1E cells. (A) 40×. Bar = 50 µm. (B) 100×. Bar = 20 µm. (C+D) DEDTC treatment for 24 hours removed most autometallographically-detectable Zn^2+^ from the INS-1E cells. (C) 40×. Bar = 50 µm. (D) 100×. Bar = 20 µm.

**Figure 3:**
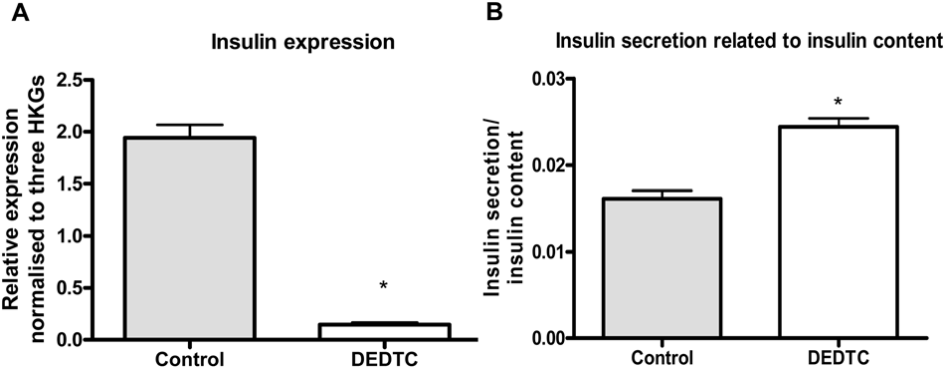
Insulin expression, content and secretion after 24 hours of 100 µM DEDTC treatment. INS-1E cells were treated with DEDTC at 5 mM glucose. A) Insulin gene expression normalized to Cltc, HPRT and HSPcb. Data are mean and SEM (*p<0.01). N = 6. B) Insulin secretion related to insulin content in INS-1E cells. Data are mean and SEM (*p<0.01). N = 3.

**Figure 4:**
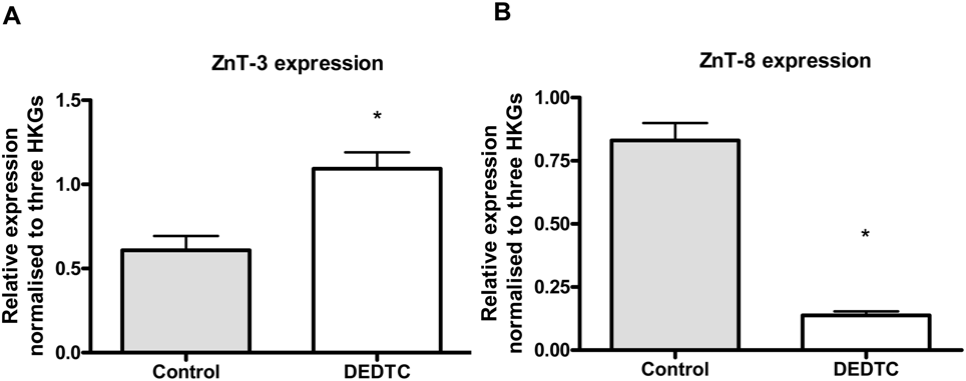
Relative gene expression of ZnT-3 and ZnT-8 after 24 hours of 100 µM DEDTC treatment. INS-1E cells were treated with DEDTC at 5 mM glucose. A) ZnT-3 gene expression normalised to Cltc, HPRT and HSPcb. Data are mean and SEM (*p<0.05). N = 6. B) ZnT-8 gene expression normalised to Cltc, HPRT and HSPcb. Data are mean and SEM (*p<0.01). N = 6.

In order to examine the effects of zinc chelation we treated INS-1E cells grown for 24 hours at 11 mM glucose with a zinc chelator (100 µM DEDTC) and visualized free intracellular zinc ions by autometallography. We found that DEDTC removed most of the detectable zinc in β-cells (Fig. 2). After 24 hours of DEDTC-treatment at 5 mM glucose we examined insulin gene expression, intracellular insulin content and insulin secretion along with the expression of ZnTs. Insulin expression decreased significantly after DEDTC treatment (p<0.01), whereas the ratio between insulin secretion and insulin content was increased in DEDTC treated cells (p<0.01) due to a significantly lower insulin content in DEDTC treated cells (data not shown) (Fig. 3A and Fig. 3B). DEDTC treatment stimulated the expression of ZnT-3 significantly (p<0.05) (Fig. 4A) but decreased the expression of ZnT-8 (p<0.01) (Fig. 4B) (all comparisons relative to untreated cells). We have previously demonstrated that ZnT-3, ZnT-8 and insulin expression varies with DEDTC treatment, and that the results depend on specific housekeeping genes [29]. Accordingly we normalised to HPRT, Cltc and HSP since they were the most stable HKGs.

### Hyperglycemia and zinc chelation induce cell death in INS-1E cells

**Figure 5:**
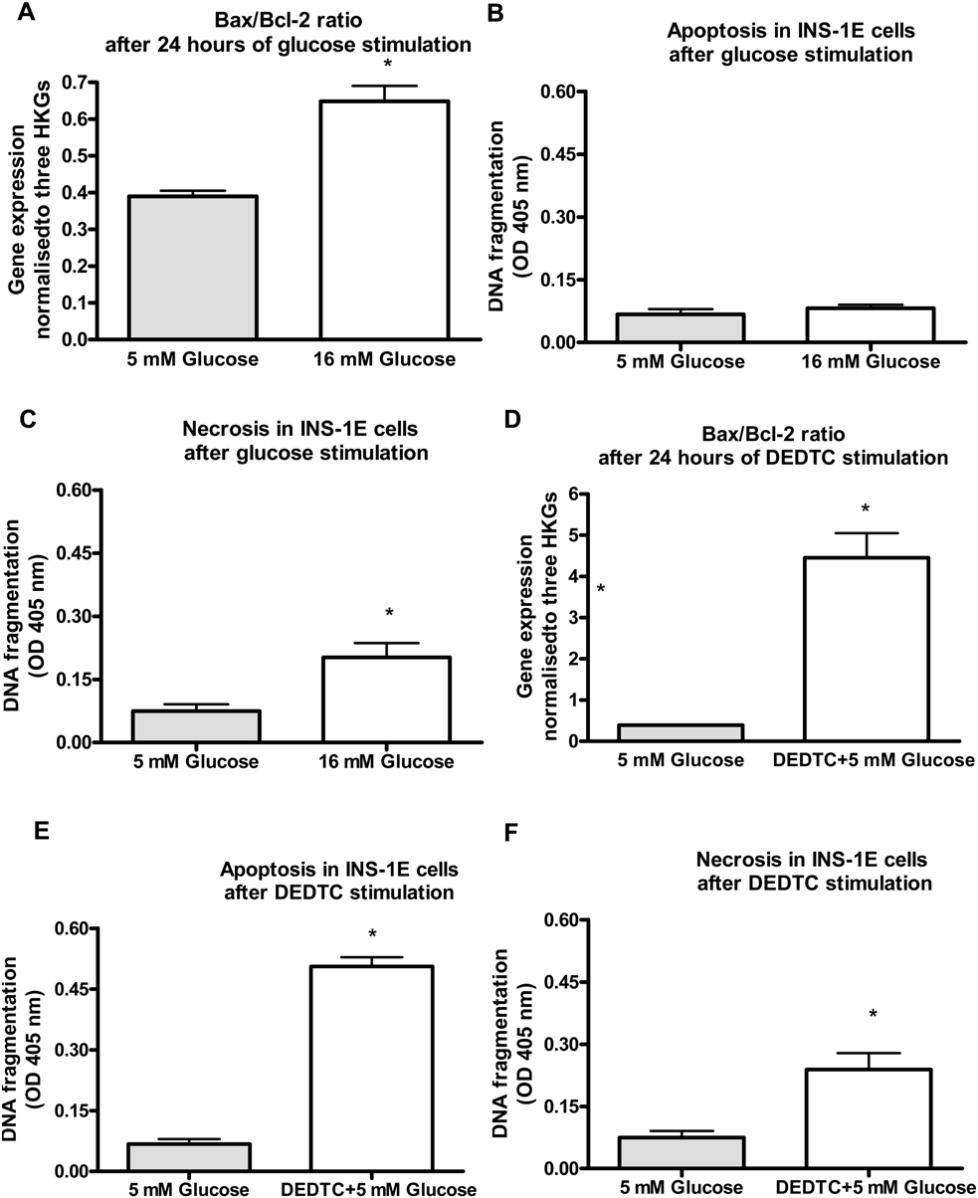
Detection of apoptosis in INS-1E cells after 24 hours of hyperglycamia or zinc depletion. A–C) Glucose stimulation with 5 mM and 16 mM. A) Bax/Bcl-2 ratio of gene expression. Both genes were normalised to Cltc, HPRT and HSPcb. Data are mean and SEM (*p<0.01). N = 6. B) Detection of intracellular DNA fragments in INS-1E cells (apoptosis) after 16 mM glucose stimulation. Data are mean and SEM. N = 4. C) Detection of DNA fragments in medium from INS-1E cells (necrosis) treated 16 mM glucose. Data are mean and SEM (*p<0.05). N = 4. D–F) Zinc chelation with 100 µM DEDTC. D) Bax/Bcl-2 ratio of gene expression. Both genes were normalised to Cltc, HPRT and HSPcb. Data are mean and SEM (*p<0.01). N = 6. E) Detection of DNA fragments in INS-1E (apoptosis) after 100 µM DEDTC treatment. Data are mean and SEM (*p<0.01) N = 4. F) Detection of DNA fragments in medium from INS-1E cells (necrosis) treated with 100 µM DEDTC. Data are mean and SEM (*p<0.01). N = 4.

The apoptotic effect of hyperglycemia and DEDTC on INS-1E cells was tested next. We analysed the gene expression of Bcl-2 and Bax. We found that hyperglycemia increased the Bax/Bcl-2 ratio (p<0.01) (Fig. 5A). Analysis of DNA fragmentation confirmed cell death, not by apoptotis but by necrosis (p<0.05) (Fig. 5B and Fig. 5C). In mouse islets hypoglycemia but not hyperglycemia increased the Bax/Bcl-2 ratio (p<0.01) (data not shown). Zinc-chelation by DEDTC in INS-1E cells lead to an up-regulation of the Bax/Bcl-2 ratio (p<0.01) (Fig. 5D) and measuring DNA fragments showed that the cells were apoptotic as well as necrotic (p<0.01 and p<0.01, respectively) (Fig. 5E and Fig. 5F). In order to avoid any effect of glucose on cell death we chose to stimulate the INS-1E cells with 5 mM glucose instead of 11 mM glucose in the DEDTC experiment because our data indicated that 11 mM tended to affect the necrotic state of INS-1E cells (data not shown).

### ZnT-3 protein is present in INS-1E cells and mouseislets

**Figure 6:**
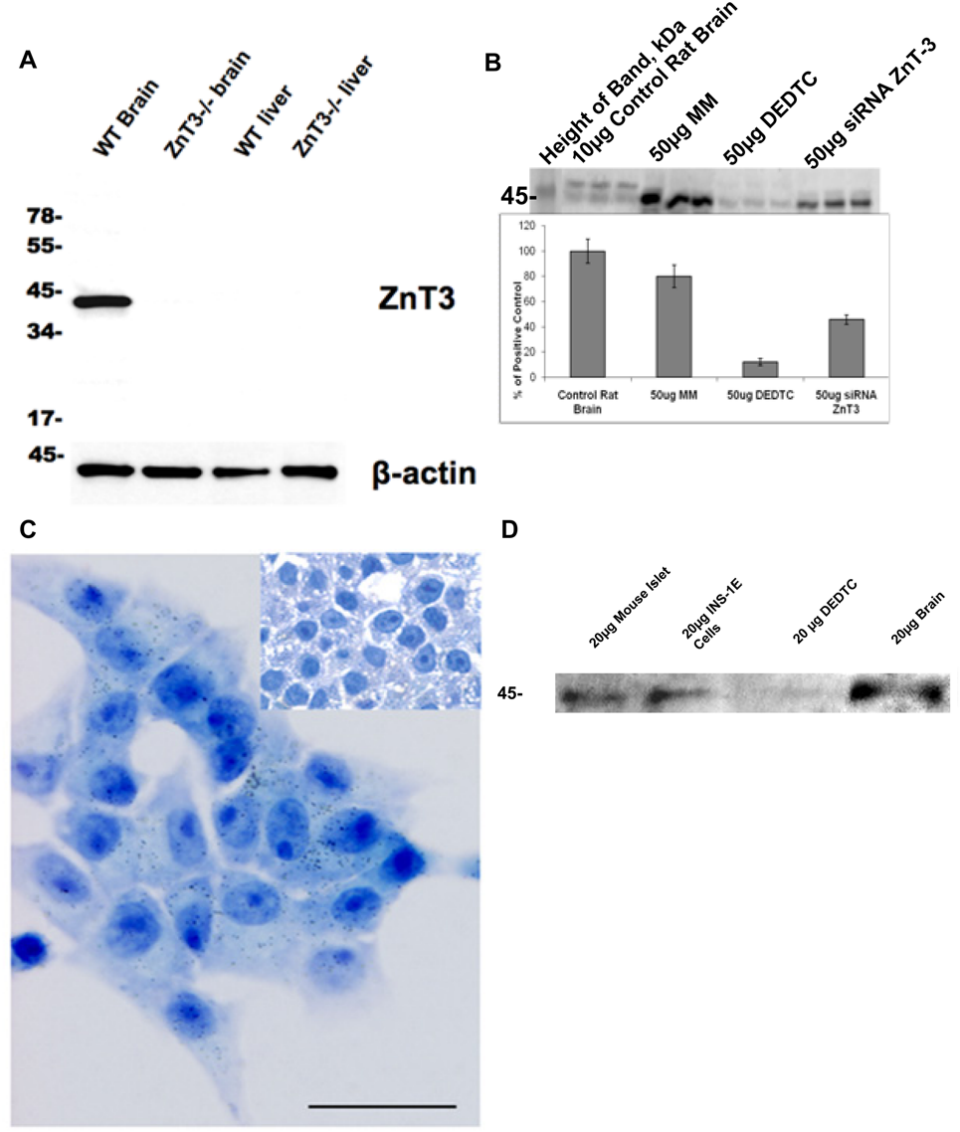
ZnT-3 protein in INS-1E cells and mouse islets. A) Western blot of ZnT-3 knockout tissue, and normal background strain tissue using the anti-ZnT-3 polyclonal antibody (20 µg per lane). B) Western blot with ZnT-3 antibody. Lane one shows the protein marker in kDa. Subsequent lanes: Control rat brain (10 µg protein) (lanes 2–4), mock transfected INS1-E cells (50 µg protein) (lanes 5–7), 100 µM DEDTC-treated INS1-E cells (50 µg protein) (lanes 8–10), ZnT-3 siRNA transfected INS1-E cells (50 µg protein) (lanes 11–13). Insert shows the quantification, brain tissue values are original multiplied by 5. C) Light micrograph of INS-1E cells exposed to ZnT-3 antibody. Silver enhanced colloidal gold (10 nm) particles attached to secondary antibodies against the ZnT-3 primary antibody are seen within the cells. There was no background stain and controls were negative (insert). Bar = 20 µm. D) Demonstration of ZnT3 antibody positivity in intact mouse islets (lane 1), compared with INS-1E cells before (lane 2) and after (lane 3) treatment with 100 µM DEDTC and brain tissue (lane 4). Each upload with 20 µg protein.

We developed a polyclonal specific antibody against the N-terminal domain of ZnT-3 (Fig. 6A). By western blotting, we determined the presence of ZnT-3 protein in DEDTC treated or siRNA transfected INS-1E cells (Fig. 6B). Brain tissue was used as control since high levels of ZnT-3 protein has previously been detected in synaptic vesicles of the hippocampus [30] (Fig. 6B). In contrast to the increased mRNA levels observed (fig. 4A), treatment of INS-1E cells with DEDTC decreasedZnT-3 protein levels assayed by western blotting (Fig. 6B). Knock-down of the ZnT-3 gene decreased protein levels by 57% (Fig. 6B). ZnT3 protein was present also in intact islets (Fig. 6D). Localisation of ZnT-3 in INS-1E cells to an intracellular compartment was determined by immunohistochemistry (Fig. 6C).

### Insulin expression and secretion is decreased by ZnT-3 knock-down in INS-1E cells

**Figure 7:**
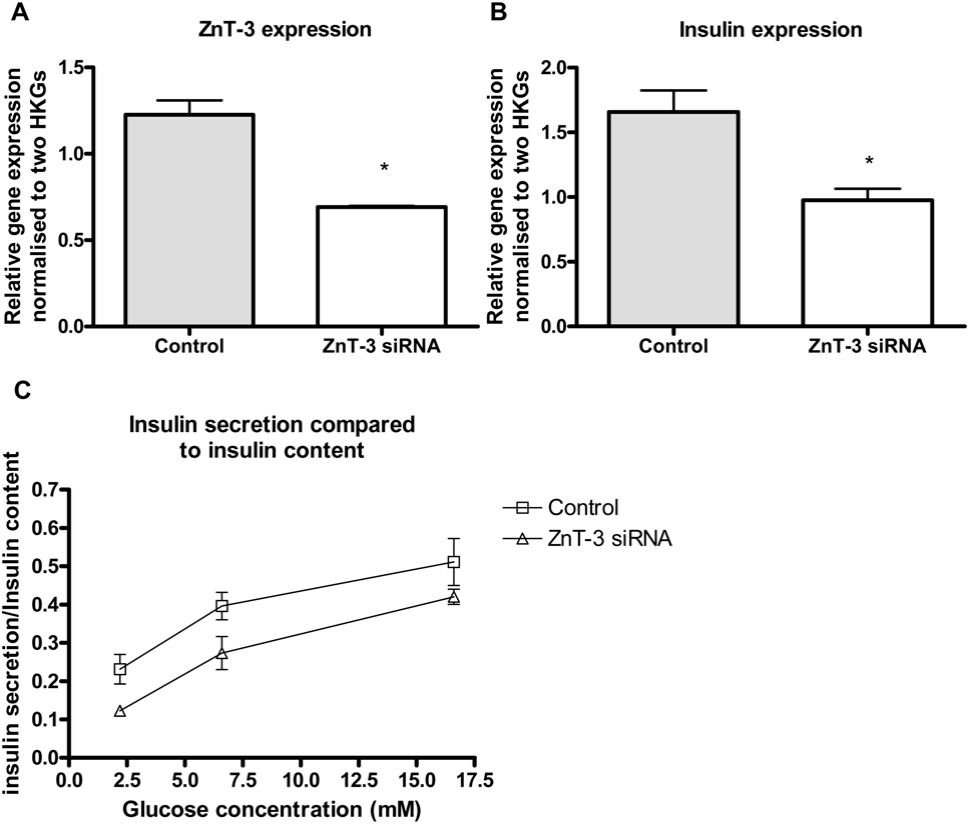
Knock-down of ZnT-3 in INS-1E cells. A) Relative gene expression of ZnT-3 in ZnT-3 knock-downed INS-1E cells compared to mismatch controls. ZnT-3 gene expression normalised to β-actin and HSP. Data are mean and SEM (*p<0.05) N = 3. B) Relative gene expression of insulin in ZnT-3 knock-down INS-1E cells. Insulin gene expression normalised to β-actin and HSP. Data are mean and SEM (*p<0.05). N = 3. C) Glucose stimulated insulin secretion in ZnT-3 knock-down INS-1E cells. Cells were stimulated with either 2, 6.6 or 16.6 mM glucose for 2 hours. Insulin secretion was normalised to insulin content. Data are mean and SEM (p<0.01 for AUC). N = 4.

To examine the *in vitro* effect of ZnT-3 knock-down we transfected INS-1E with ZnT-3 siRNA. The ZnT-3 mRNA level was knocked down by 44% (p<0.05) and insulin expression was reduced by 41% (p<0.05) (Fig. 7A and Fig. 7B). AUC for insulin secretion/insulin content was decreased in ZnT-3 siRNA transfected INS-1E cell compared to mock-transfected INS-1E cells (p<0.05) (Fig. 7C).

### Glucose metabolism is impaired in ZnT-3 knock-out mice

**Figure 8:**
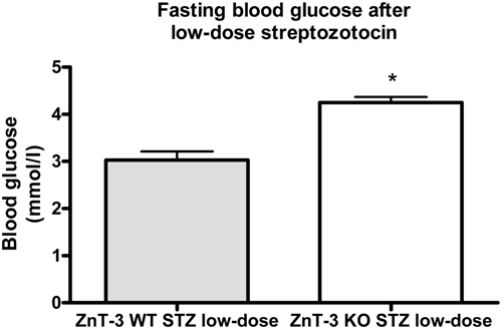
Fasting glucose levels after low-dose streptozotozin in ZnT-3−/− knock-out mice. ZnT-3−/− knock-out mice and control mice were treated with 50 mg/kg/day for five days. Data are mean and SEM (*p<0.01). N = 15.

**Figure 9:**
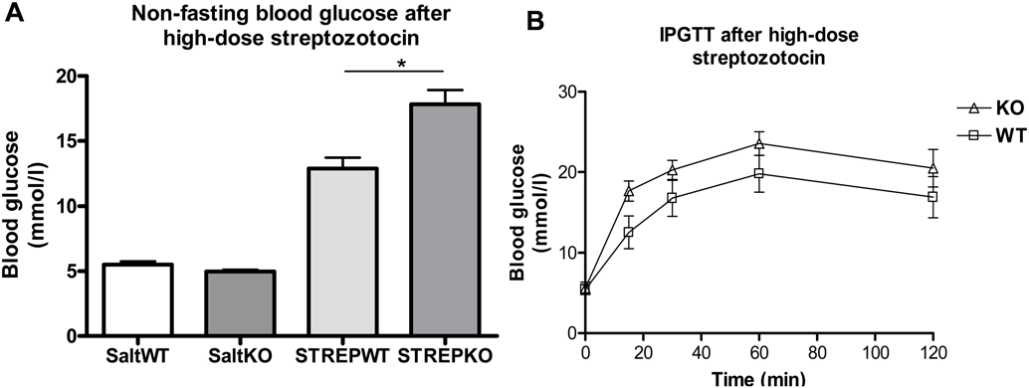
High-dose streptozotocin for three days in ZnT-3−/− knockout mice and control mice. Following a series of low-dose exposures (see Fig. 8) ZnT-3−/− knock-out mice and control mice were treated with 200 mg/kg/day. A) Non-fasting morning blood glucose levels. Salt indicates sham saline injections. Data are mean and SEM (*p<0.01). B) Intraperitoneal glucose tolerance test in ZnT-3−/− knockout mice and control mice after high-dose streptozotozin. Blood glucose concentrations were measured before and 15, 30, 60 and 120 minutes after the glucose injection. Significantly higher AUC_0–120_ in knock-out vs WT mice (p<0.01). N = 5 for ZnT-3−/− and n = 4 for wild type.

Knocking out ZnT-3 affected *in vivo* glucose metabolism after low- and high-dose streptozotocin, most significantly following severe β-cell stress. There were no significant differences in body weights when comparing knock-out and wild-type mice. Fasting blood glucose levels were unaffected by ZnT-3 knock-out, data not shown, thus under normal circumstances glucose metabolism after knock down resembles that of the wild-type. After a single series of low-dose streptozotozin (50 mg/kg/day for 5 days) all mice, knock-out and wild-type, remained non-diabetic with a slight increase in fasting glucose levels in the knock-out group (p<0.01) (Fig. 8). IPGTTs showed no difference when measuring AUC for glucose. Repeated courses of low-dose streptozotocin did not cause diabetes in either group and their IPGTTs were similar. High-dose streptozotocin (200 mg/kg/day for 3 days) resulted in significantly higher glucose levels in the knock-out group both while non-fasting and after IPGTT (p<0.01 for both comparisons) (Fig. 9).

## Discussion

A number of observations support the importance of zinc in maintaining a normal glucose homeostasis. Low amounts of free (non-enzymatic) zinc are associated with a decreased insulin content in β-cells [31] and zinc depletion by DEDTC leads to diabetes [32], [33]. Recently, Gyulkhandanyan et al. [34] demonstrated in mouse β-cells that zinc transport from the extracellular space into the cytoplasm is regulated by the activity of L-type calcium channels. During high-glucose exposure L-type calcium channels appear to be crucial and in resting states ZIP proteins gain responsibility for the inward zinc transport. On the other hand, ZnTs are responsible for the outward transport i.e. from cytoplasm to extracellular spaces or intracellular vesicles. The transport of zinc into secretory vesicles is vital for the crystallization of insulin [2].

We here describe that ZnT-1 and ZnT-3-8 genes are expressed in INS-1E cells. We studied the expression of zinc transporters in response to either different levels of glucose or removal of non-protein-bound zinc by DEDTC. At high glucose concentrations, ZnT-8 expression was down-regulated whereas ZnT-3 expression was up-regulated. In mouse islets ZnT-3 expression was up-regulated by high glucose concentrations. This is the first demonstration that glucose can significantly influence the gene expression of zinc transporting proteins.

Previously, we have shown that treatment with 5 mM DEDTC removes all non-protein-bound zinc in INS-1E cells when treated for 30 minutes [26]. In order to achieve a long-term chelation of zinc we treated INS-1E cells with 100 µM DEDTC for 24 hours. This led to the removal of the majority of the zinc. ZnT-8 and insulin expressions were down-regulated after DEDTC treatment while ZnT-3 gene expression increased and protein levels decreased after DEDTC. Thus, zinc depletion as well as high glucose concentrations appears to affect the expression of ZnT-3 and ZnT-8 in opposite directions, indicating that these two transporters in particular play a pivotal role in maintaining β-cell zinc homeostasis.

Stimulation with high glucose leads to glucotoxicity in human pancreatic islets causing β-cell death [35]. In our study, Bcl-2 and Bax gene expressions were indicative of cell death after stimulation with high glucose and after treatment with DEDTC in INS-1E cells. These results were confirmed by an ELISA based cell death detection kit. In mouse islets, 24 hour of stimulation with high glucose concentrations did not lead to an increased Bax/Bcl-2 ratio, indicating that glucotoxicity arises at a later time than 24 hours in primary cells.

ZnT-8 over-expressing INS-1E cells are protected from zinc depletion-induced cell death compared to control cells and ZnT-8 over-expression leads to increased glucose-induced insulin secretion [36]. Our study shows that the benefits of ZnT-8 on the cell survival and the insulin secretion may be absent in non-transfected cells due to a glucose or DEDTC mediated down-regulation of ZnT-8 expression. We found that ZnT-3 is up-regulated by high glucose.

ZnT-3, which thus appears to be responsive to both glucose and zinc depletion in β-cells, is known to be responsible for the transport of zinc into synaptic vesicles in the hippocampus and cerebellum, both regions rich in neurones using zinc for neuromodulation and ZnT3 may be a marker for apoptosis [37], [38], [39]. Removal of ZnT-3 results in defective neurotransmission and cytoplasmic accumulation of potentially toxic zinc ions [18], [40]. Expression levels may be influenced by exogenous stimuli other than glucose and DEDTC, e.g. oestrogen lowers ZnT-3 expression in mouse brain [41]. ZnT-3 is an important factor in AD since ZnT-3 is supplying the zinc which is partly responsible for the formation of amyloid plaques [13]. It may be hypothesized that the link between ZnT-3 and amyloid-related pathologies, such as type 2 diabetes, is present in β-cells as well as in the hippocampus.

ZnT-3 has not previously been detected in β-cells [42]. We here demonstrate the presence of ZnT-3 protein in INS-1E cells and the decrease of protein levels after ZnT3 gene knock-down.

By contrast, ZnT-8 has previously been shown to be present in β-cells, located in secretory vesicles. Our current observations suggest ZnT-3 to be another key player. Using ZnT-3 knock-down INS-1E cells we were able to reveal *in vitro* the effects of down-regulation of the ZnT-3 gene on insulin expression and secretion. Using a knock-out mouse model, we demonstrated that the *in vivo* glucose metabolism can be affected by alterations in the zinc transporter system. Since this was evident only after severe β-cell stress, compensatory mechanisms must exist, the maintenance of intracellular and intravesicular zinc levels in β-cells is most likely dependent upon a number of interacting transporters. The impact of changes in the expression of zinc transporters on other actions of zinc in islets (most notably regulation of β-cell mass and glucagon secretion) remains to be settled [7].

In conclusion, we have found that a number of ZnTs are present in β-cells. ZnT-3 expression is up-regulated by glucose and zinc depletion whereas ZnT-8 is down-regulated. Zinc depletion decreased protein levels. This is the first evidence that zinc transporting proteins are actively influenced by the metabolic status of β-cells and suggests that β-cell zinc homeostasis must be further studied in order to establish whether this system has a role also in β-cell pathologies. In the ZnT-3 knock-out model we have demonstrated that *in vivo* glucose metabolism can be affected by alterations in zinc transporters. This was also suggested by large-scale epidemiological studies in populations of patients [8] and β-cell zinc metabolism is emerging as yet another important system in understanding the development of type 2 diabetes.
